# Implementing log file‐based patient‐specific QA for VMAT plans: A comparative study of MobiusFX and measurement‐based approaches

**DOI:** 10.1002/acm2.70526

**Published:** 2026-02-24

**Authors:** Thitipong Sawapabmongkon, Pimolpun Changkaew, Chanon Puttanawarut, Puangpen Tangboonduangjit, Suphalak Khachonkham

**Affiliations:** ^1^ Division of Radiation Oncology Department of Diagnostic and Therapeutic Radiology Faculty of Medicine Ramathibodi Hospital Mahidol University Bangkok Thailand; ^2^ Chakri Naruebodindra Medical Institute Faculty of Medicine Ramathibodi Hospital Mahidol University Samut Prakan Thailand

**Keywords:** log file‐based PSQA, MobiusFX, patient‐specific QA, VMAT

## Abstract

**Background:**

The current interest in utilizing log file‐based patient‐specific quality assurance (PSQA) is growing as an alternative to measurement‐based approaches for volumetric modulated arc therapy (VMAT) plans. However, implementing log file‐based PSQA requires further experience and understanding since there are no guidelines.

**Purpose:**

This study aimed to evaluate the feasibility of implementing commercial log file‐based PSQA (MobiusFX) for pretreatment verification of VMAT plans by comparing its outcomes with measurement‐based methods.

**Methods:**

The study was conducted by selecting 40 VMAT plans and these plans were performed for pretreatment verification for both MobiusFX and measurement‐based methods, including portal dosimetry, ArcCHECK, and Octavius4D with gamma analysis at criteria 3%/3 mm, 3%/2 mm, and 2%/2 mm. The agreement of gamma passing rate between MobiusFX and measurement‐based methods was assessed through scatter plots and percentage agreement score. Additionally, the root mean square (RMS) errors obtained from MobiusFX were investigated in each machine parameter, and examined the correlation between the gamma passing rate and plan complexity metric (MU/Gy) by Spearman's rank correlation coefficient. The statistical control process concept from TG 218 was also applied to establish the action limit (AL) and tolerance limit (TL) for MobiusFX.

**Results:**

The scatter plots of MobiusFX illustrated a trend similar to the measurement‐based methods, with high percentage agreement scores observed at 3%/3 mm and 3%/2 mm criteria. RMS errors were zero for all parameters except multileaf collimator (MLC), and the maximum RMS was 0.06 mm. No correlation was found between the gamma passing rate and the complexity metric in this study. The most appropriate TL and AL for the gamma passing rate with MobiusFX were determined to be 95.38% and 94.75%, respectively, at the 3%/2 mm criterion.

**Conclusion:**

MobiusFX showed interchangeable gamma passing rates with measurement‐based methods at the 3%/3 mm and 3%/2 mm criteria, supporting its use as an alternative for VMAT pretreatment verification. Machine‐ and system‐specific factors should be considered for reliable clinical implementation.

## 1 INTRODUCTION

A sophisticated treatment delivery technique, volumetric modulated arc therapy (VMAT), has been widely utilized in numerous radiation oncology clinics for cancer treatment. The aforementioned technique offers high conformity while effectively sparing normal tissue through modulation parameters, which include multileaf collimator (MLC) movement, gantry rotation, and dose rate.^1^, ^2^, ^3^ As the technique becomes more advanced and complex for linear accelerator (linac) machine delivery, the chances of errors occurring also increase. The occurrence of these errors might lead to overdosing and underdosing, which can worsen the progression of treatment more than expected. Furthermore, it could unintentionally lead to a lethal radiation overdose, as reported in a New York Times article in 2010.^4^ To minimize the occurrence of harmful accidents during the course of treatment, it is crucial to detect these errors prior to patient treatment. Therefore, patient‐specific quality assurance (PSQA) is introduced as the necessary procedure before radiation treatment delivery to confirm the accuracy of individual patient's treatment plan for patient safety.^5^, ^6^

Traditionally, PSQA has relied on measurement‐based methods such as ionization chambers, detector arrays, and film to verify the calculated dose from the treatment planning system (TPS) against the delivered dose distribution. However, these methods are notably labor‐intensive and considerably increase the clinical workload. Consequently, there is growing interest in log file‐based PSQA as an alternative approach that eliminates the need for setting up physical phantoms or additional hardware, resulting in a less time‐consuming process.^7^, ^8^, ^9^, ^10^ In addition, several studies highlight that log file‐based PSQA is superior in certain aspects to traditional measurement‐based methods. In particular, log file‐based approaches can detect certain errors such as plan transfer discrepancies and human‐related operational errors that are undetectable by measurement‐based methods.^11^ Furthermore, previous studies indicate that log file‐based PSQA exhibits higher sensitivity in identifying subtle machine discrepancies.^12^, ^13^

By definition, log files contain both the actual and expected values of machine parameters for the linac during the beam delivery process, including MLC movement, gantry angle, collimator position, and others. Due to their high level of detail, these parameters have been utilized in the development of log file‐based PSQA in several studies.^11^, ^14^, ^15^ For example, Rangaraj et al. implemented in‐house QA software based on log files to assess the delivered machine parameters. This was achieved by comparing the delivered parameters of gantry, collimator, and jaw positions, as obtained from the log file, with the planned values in each patient's treatment plan at their institution. Furthermore, the MLC positions were extracted from log file to regenerate delivered fluence intensity and compare it with planned fluence intensity.^11^ Similarly, Defoor et al. used the recorded MLC patterns retrieved from the log file, which cooperated with the algorithm for dose calculation, to obtain the dose distribution and perform gamma analysis.^15^ Moreover, log file‐based PSQA is highly suitable for verifying the treatment plan in adaptive radiotherapy, where PSQA is challenging. During treatment, the patient remains on the couch while the modified plan requires immediate verification. When the adapted plan is delivered, log file data is generated, enabling rapid plan assessment without patient repositioning or reliance on measurement‐based QA.^16^

Currently, log file‐based PSQA is commercially available as part of the Mobius3D (Varian Medical Systems Inc., Palo Alto, CA, USA), a software package designed for computational‐based PSQA. This software includes an add‐on module called MobiusFX, which serves as an additional part that further enhances the capabilities of the software. This module automatically verifies the delivered 3D dose distribution by utilizing the dose calculation algorithm together with delivered machine parameters from the log file. The outcomes can be compared with the TPS through various analyses, including gamma analysis, mean target dose difference, and dose‐volume histogram (DVH) for each fraction. Previous studies have found that MobiusFX has the capability to produce comparable outcomes to measurement‐based approaches in PSQA.^17^, ^18^, ^19^, ^20^, ^21^ These findings pointed out the feasibility of using MobiusFX as an alternative approach for pretreatment verification. This method is becoming increasingly interesting because it eliminates the need for dosimetry devices during the setup process and reduces the workload for PSQA.

Despite log file‐based PSQA having numerous beneficial characteristics and fascinating properties, ongoing discussions take place among the medical physicist community regarding the replacement of measurement‐based methods with log file‐based approaches.^10^ There are concerns regarding the reliability of log file‐based PSQA in some issues. A previous study revealed that loose T‐nuts connecting the motor to the MLC leaves were related to recording errors in log files.^22^ Another concern has been raised about dosimetric parameters, such as output variations, that cannot be recorded in the log file.^18^ Most importantly, there is a lack of consensus regarding the criteria and guidelines for log file‐based PSQA. Therefore, further experience and understanding are still needed to effectively utilize log file‐based PSQA. Following these controversial issues, this study aimed to explore the possibility of comprehensively implementing MobiusFX by comparing its outcomes with measurement‐based methods, emphasizing gamma passing rate agreement, RMS machine errors, as well as the establishment of action and tolerance limits, in order to support the feasibility of using the log file‐based method as an alternative approach for pretreatment verification QA.

## 2 MATERIALS AND METHODS

### 2.1 Overview of research design

This study was conducted retrospectively. A total of 40 VMAT treatment plans delivered using the TrueBeam linear accelerator (Varian Inc., Palo Alto, CA) between May 2021 and August 2022 were randomly selected for analysis. This study was approved by the Institutional Review Board (IRB) (MURA2022/662). The selected plans, including 11 head and neck plans, 14 pelvis plans, and 15 chest plans, were utilized to create pretreatment verification plans through Eclipse TPS (version 16.1.0, Varian Medical Systems, Palo Alto, CA) with the analytical anisotropic algorithm (AAA). The details of each plan are described in Table 1. These plans were generated for PSQA through both log file‐based and measurement‐based methods. The log file‐based PSQA was performed using the MobiusFX module in the Mobius3D, while measurement‐based methods utilized the electronic portal imaging device (EPID; a‐Si 1200, Varian Medical Systems, Palo Alto, CA), cylindrical diode array detector (ArcCHECK; Sun Nuclear Corporation, Melbourne, FL, USA), and ionization chamber array detector (Octavius4D; PTW‐Freiburg, Freiburg, Germany). Gamma analysis was conducted in each system, following the concept from Low et al.^23^ Several data analyses were conducted to investigate the results and compared the outcomes between measurement‐based and log file‐based methods.

**TABLE 1:** The details of each plan including treatment site, energy, technique, number of arcs, and complexity metric (MU/Gy).

Case number	Treatment site	Energy (MV)	Technique	No. Arc	MU/Gy
1	Head and neck	6X	VMAT	3	210.5
2	Head and neck	6X	VMAT	3	258.5
3	Head and neck	6X	VMAT	3	206.0
4	Head and neck	6X	VMAT	3	334.5
5	Head and neck	6X	VMAT	2	236.5
6	Head and neck	6X	VMAT	3	290.5
7	Head and neck	6X	VMAT	3	232.0
8	Head and neck	6X	VMAT	3	232.2
9	Head and neck	6X	VMAT	3	266.5
10	Head and neck	6X	VMAT	3	262.5
11	Head and neck	6X	VMAT	3	310.4
12	Pelvis region	6X	VMAT	3	217.0
13	Pelvis region	6X	VMAT	3	361.7
14	Pelvis region	6X FFF	VMAT	3	473.7
15	Pelvis region	6X FFF	VMAT	3	383.8
16	Pelvis region	10X	VMAT	3	317.6
17	Pelvis region	10X	VMAT	3	257.0
18	Pelvis region	10X	VMAT	3	320.6
19	Pelvis region	10X	VMAT	3	317.1
20	Pelvis region	6X	VMAT	3	258.3
21	Pelvis region	6X	VMAT	3	340.0
22	Pelvis region	6X	VMAT	3	356.6
23	Pelvis region	6X FFF	VMAT	2	237.7
24	Pelvis region	6X	VMAT	2	268.0
25	Pelvis region	6X	VMAT	3	429.4
26	Chest region (lung)	6X	VMAT	3	381.5
27	Chest region (lung)	6X	VMAT	4	379.0
28	Chest region (lung)	6X FFF	VMAT	3	249.5
29	Chest region (lung)	6X	VMAT	3	332.5
30	Chest region (lung)	6X	VMAT	3	296.0
31	Chest region (lung)	6X	VMAT	2	250.0
32	Chest region (lung)	6X FFF	VMAT	3	294.9
33	Chest region (lung)	6X	VMAT	4	592.0
34	Chest region (lung)	10X	VMAT	3	229.5
35	Chest region (Breast)	6X	VMAT	3	256.5
36	Chest region (Breast)	6X FFF	VMAT	3	214.7
37	Chest region (Breast)	6X	VMAT	3	290.7
38	Chest region (Breast)	6X	VMAT	3	429.4
39	Chest region (lung)	6X	VMAT	3	258.0
40	Chest region (Breast)	6X	VMAT	2	349.5

### 2.2 Mobius3D software and MobiusFX (log file‐based PSQA)

Mobius3D (version 3.1) is a commercial software for computational‐based PSQA that provides comprehensive QA functions. The main component of Mobius3D software includes both a dose calculation algorithm, collapsed cone convolution superposition (CCCS), and beam model for calculating 3D dose distribution. The beam model was generated for the TrueBeam linac at each energy using reference data provided by the vendor. The Mobius3D system includes three function modules: MobiusCalc, MobiusFX, and MobiusCB. For this study, only MobiusCalc and MobiusFX were utilized to conduct the experiment. MobiusCalc served as the secondary dose check system, while MobiusFX was used for log file‐based PSQA.

Prior to starting this study, the Mobius3D software carefully underwent a complete commissioning process for the TrueBeam linac, following the procedures suggested by the vendor.^24^ The modeled Mobius3D parameters, including off‐axis ratio (OAR), percentage depth dose (PDD), and output factor, closely matched the measurement data with percentage differences of 2.0%, 1.5%, and 0.5%, respectively, for all energies. These recommended percentage differences are suggested by the vendor. Additionally, the dosimetric leaf gap (DLG) correction factor can be adjusted in order to account for the DLG effect between the linac machine and software configuration. This factor was also optimally adjusted for each energy.^25^

**FIGURE 1:**
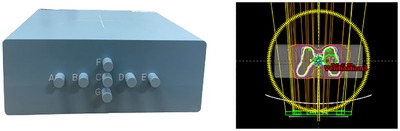
The MVP and the creation of a verification plan in Eclipse TPS.

To prepare log file‐based PSQA, the 40 VMAT plans were used to generate verification plans on a mobius verification phantom (MVP), which is a homogeneous water‐equivalent material phantom (Figure 1). Subsequently, these plans were sent from Eclipse TPS to the Mobius3D software through DICOM‐RT files which include datasets of computed tomography (CT) images, RT dose, RT plan, and RT structure. These data were utilized to independently recalculate and generate the 3D dose distributions using the CCCS algorithm and the Mobius3D beam model in the software configuration. In this study, both the TPS and Mobius3D used the same voxel size of 2.5 mm for dose calculation. This process was conducted using the MobiusCalc module, which serves as the secondary dose check system.

After the calculation process was fully completed in the MobiusCalc module, the pretreatment verification plans were then delivered via the TrueBeam linac. During the delivery process, the expected and actual machine parameters were recorded in the log file and automatically transferred to the Mobius3D software. The delivered machine parameters collaborated with the previously calculated information in the MobiusCalc module for each plan to generate the delivered 3D dose distribution. The dose distribution was subsequently evaluated by comparing it with the calculated dose from the TPS and conducting a 3D gamma analysis. This process was conducted using the MobiusFX module, which serves as the log file‐based method. To replicate the workflow of measurement‐based method and detect errors before delivering the plan to the patient, this study utilized MobiusFX for pretreatment verification by delivering the treatment plan prior to patient irradiation (fraction 0).

MobiusFX is different from measurement‐based approaches which can comprehensively display not only 3D gamma analysis but also root mean square (RMS) error in each delivered machine parameter. The RMS error was determined as:

(1)

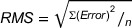




In the equation 1, the error is determined by the difference between the delivered and expected machine parameters, extracted from the log file. These differences are squared, summed over all control points, divided by the overall count of control points (*n*), and then the square root is taken to determine the RMS error in MobiusFX. The RMS error was calculated for various parameters, including the jaw positions (*X*1, *X*2, *Y*1, and *Y*2), collimator angle, gantry angle, and MLC positions (both bank A and bank B). The RMS errors obtained from the aforementioned parameters were utilized for analysis in the section below.

### 2.3 EPID‐based dosimetry

The EPID detector is an array of amorphous silicon diodes that is attached to the TrueBeam linac arm, with a maximum detector dimension of 43 × 43 cm^2^. The matrix size is 1280 × 1280 pixels, and the pixel size is 0.336 mm. The pretreatment verification plan was created through the portal dosimetry feature in the Eclipse TPS. Before performing PSQA, an absolute dose calibration procedure was conducted to scale 100 MU to 1 calibrated unit (CU) delivered by a field size of 10 × 10 cm^2^ with a source‐to‐detector distance of 100 cm in each energy. Furthermore, dark field and flood field calibrations were performed to correct the background and sensitivity in each pixel, respectively.^26^, ^27^ To perform gamma analysis, the PSQA results were also analyzed through the portal dosimetry feature in the TPS.

### 2.4 Cylindrical diode array detector (ArcCHECK)

The ArcCHECK, a cylindrical‐shaped phantom designed with a spiral pattern arrangement of 1386 diode detectors, was utilized in this study. Each detector has an active size of 0.8 mm × 0.8 mm, with a detector spacing of 1 cm along the length of the phantom. The active region of the phantom is 21 cm in length, and the diameter of the array is 26.6 cm. Each detector in the spiral arrangement allows for the measurement of the dose at the entry and exit points along the radiation path to reconstruct the dose distribution. In order to achieve a uniform density over the detector, a homogenous RW3 plug was selected and placed in the center of the ArcCHECK.

The ArcCHECK virtual CT image set was provided by the vendor and imported into the TPS for dose calculation in each verification plan. The physical density of the phantom body was overridden to approximately 1.045 g/cm^3^. For data acquisition and analysis, SNC software version 6.2.1 was used. Before measurement procedure, a background correction was automatically carried out in SNC software. Additionally, an array calibration was also created and provided by vendor for reducing the different response in each detector. Furthermore, each energy underwent absolute dose calibration, where the measured dose was compared with the calculated dose from the TPS.^28^ After preparation processes, PSQA was conducted. The measured dose in each plan was evaluated against the calculated dose using gamma analysis through the SNC software.

### 2.5 Ionization chamber array detector (Octavius4D)

The Octavius detector 1500 (PTW‐Freiburg, Freiburg, Germany) is a 2D detector array that consists of 1405 vented ionization chambers with a spacing of 10 mm center‐to‐center. The maximum field size for this detector is 27 × 27 cm^2^. This 2D detector was inserted into the slot of the cylindrical‐shaped Octavius4D phantom, made from polystyrene material. The phantom can rotate perpendicularly with the incident beam to avoid angular dependence effects.

For dose calculation, the artificial CT image of the Octavius4D phantom was provided by the vendor and imported into the TPS for calculation in each verification plan. The relative electron density of the phantom body was overridden to approximately 1.016. Prior to the measurement procedure, a background correction or zeroing was automatically performed, and a pre‐irradiation was carried out to achieve a stable detector response. Additionally, a cross‐calibration was conducted by delivering a known dose to the detector isocenter. The measured dose and calculated dose from the TPS were utilized to determine the cross‐calibration factor for each energy.^29^ After completing these procedures, PSQA was carried out. A comparison between the TPS‐calculated and measured doses in each plan was performed using gamma analysis through the PTW Verisoft software.

### 2.6 Data collection and analyses

For both log file‐based and measurement‐based methods, the gamma analysis was carried out using 3%/3 mm, 3%/2 mm, and 2%/2 mm criteria with a 10% low‐dose threshold. The 3%/3 mm and 3%/2 mm criteria are common criteria for measurement‐based PSQA, as recommended by TG 119 and TG 218.^6^, ^30^ Meanwhile, the 2%/2 mm criterion was introduced in this study to investigate the tendency of the gamma passing rate under a stricter criterion. Additionally, the RMS error of each machine parameter was collected and analyzed from log file‐based data, which is not accessible through the measurement‐based method.

To explore the feasibility of using the log file‐based method (MobiusFX) as an alternative approach for pretreatment verification QA, the QA results from MobiusFX were investigated and compared against measurement‐based methods across the following three categories.

#### 2.6.1 Determination of agreement by employing TG 218′s action and tolerance limits

Commonly, action limit (AL) and tolerance limit (TL) for the gamma passing rate are utilized to decide whether to treat the patient or not. According to TG 218 recommendations, the universal AL and TL for the gamma passing rate, using the 3%/2 mm criterion, should exceed 90% and 95%, respectively.^6^ This section implemented the universal AL and TL to determine passing and failing for all gamma criteria (3%/3 mm, 3%/2 mm, and 2%/2 mm) in each plan. To assess the level of agreement between the log file‐based and measurement‐based methods, we plotted the gamma passing rates from both methods against the universal AL and TL. In addition, a percentage agreement score was calculated to evaluate the agreement in quantity value. The percentage agreement score was obtained by counting the plans that produced matching outcomes (either pass or fail the AL and TL) between log file‐based and measurement‐based methods. In mathematical terms, it can be defined as equation 2

(2) $$\begin{array}{cc} & \mathrm{Percentage}\;\mathrm{agreement}\;\mathrm{score}\;\\ & \;=\frac{\mathrm{number}\;\mathrm{of}\;\mathrm{agreement}\;\mathrm{plans}\;}{\mathrm{total}\;\mathrm{number}\;\mathrm{of}\;\mathrm{plans}}\times \;100 \end{array}$$

#### 2.6.2 Investigation of RMS error and plan complexity

In previous studies, it was revealed that utilizing not only gamma analysis but also the RMS error criteria for log file‐based PSQA can enhance the capability to detect and identify the source of machine errors.^12^, ^13^ This finding motivated the current study to investigate the RMS errors obtained from MobiusFX and examine the correlation between the gamma passing rate and plan complexity metric using Spearman's rank correlation coefficients. Scales for interpreting the correlation coefficient (*r*) are as follows: 0.00–0.10 (negligible), 0.10–0.39 (weak), 0.40–0.69 (moderate), 0.70–0.89 (strong), and 0.90–1.00 (very strong).^31^The plan complexity metric was determined as MU/Gy, which is obtained by dividing the total MU in a single fraction by the prescribed dose in the same fraction (Gy). It was collected individually for each plan. This metric is based on the concept that complex plans tend to consist of a higher proportion of small MLC aperture openings and a larger number of MUs.^32^, ^33^

#### 2.6.3 Establishing log file‐based PSQA action and tolerance limits using TG 218 concept

The AL and TL for gamma passing rate may vary across institutions, depending on local parameters such as specific processes, equipment, experience, and case types. Statistical process control can be specifically employed to calculate the local AL and TL for each institution, as suggested by TG 218.^6^ The local AL and TL are determined using the following equations 3 and 4, respectively.

(3) $$Action\;limit\;\;\left(AL\right)=100-\;\frac{\beta \;\sqrt{\sigma^{2}+{\left(\bar{X\;}-T\right)}^{2}}}{2}$$

where, β is the constant that equals 6 (recommended by TG 218)

*x̅* is the mean value

*σ* *2* is the variance

*T* is the target value (100% for gamma passing rate)

(4) $$\begin{array}{cc} & Tolerance\;limit\;(TL)\\ & \;=\bar{x}-2.660\left(\frac{1}{\left(n-1\right)}\sum_{i=2}^{n}\left|x_{i}-x_{i-1}\right|\right) \end{array}$$

where, *x̅* is the mean value *n* is the total number of plans *x* is the gamma passing rate currently, the log file‐based method lacks universally established AL and TL values. Therefore, in this section, we applied the statistical approach described above to determine AL and TL for the log file‐based method under each criterion and compared these values with those from the measurement‐based methods.

## 3 RESULTS

### 3.1 Determination of agreement by employing TG 218′s action and tolerance limits

**FIGURE 2:**
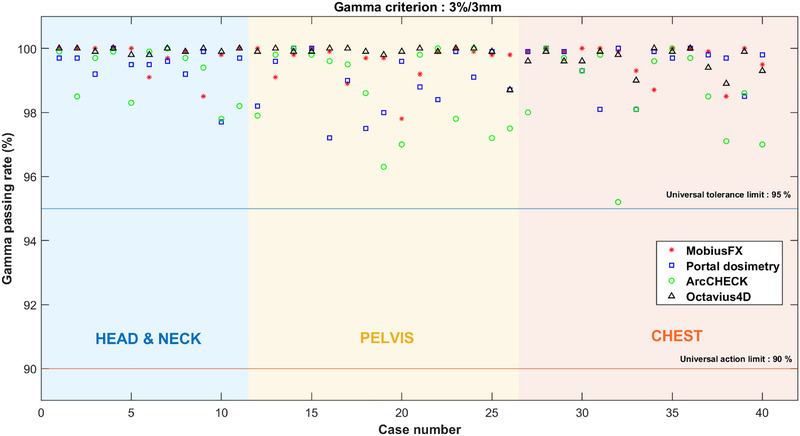
Comparison gamma passing rate across systems using the 3%/3 mm criterion. (The red line indicates the universal AL, and the blue line indicates the TL.) The gamma passing rate for each plan is represented using the following symbols: the asterisk (*) for MobiusFX, a square (□) for Portal dosimetry, a circle (°) for ArcCHECK, and a triangle (△) for Octavius4D, respectively.

**FIGURE 3:**
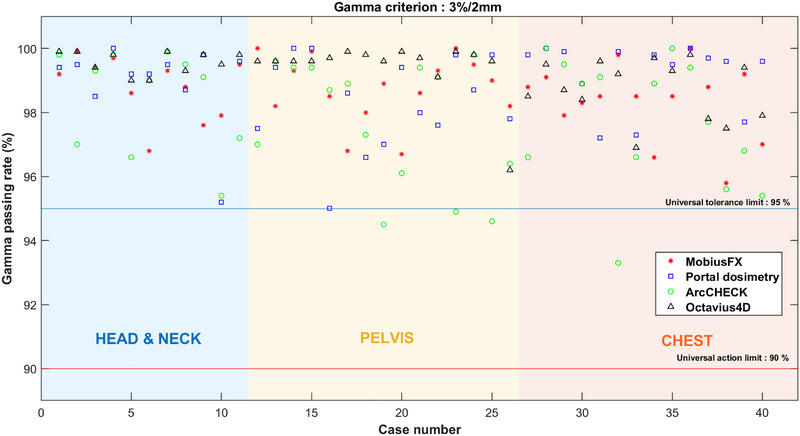
Comparison gamma passing rate across systems using the 3%/2 mm criterion. (The red line indicates the universal AL, and the blue line indicates the TL.) The gamma passing rate for each plan is represented using the following symbols: the asterisk (*) for MobiusFX, a square (□) for portal dosimetry, a circle (°) for ArcCHECK, and a triangle (△) for Octavius4D, respectively.

**FIGURE 4:**
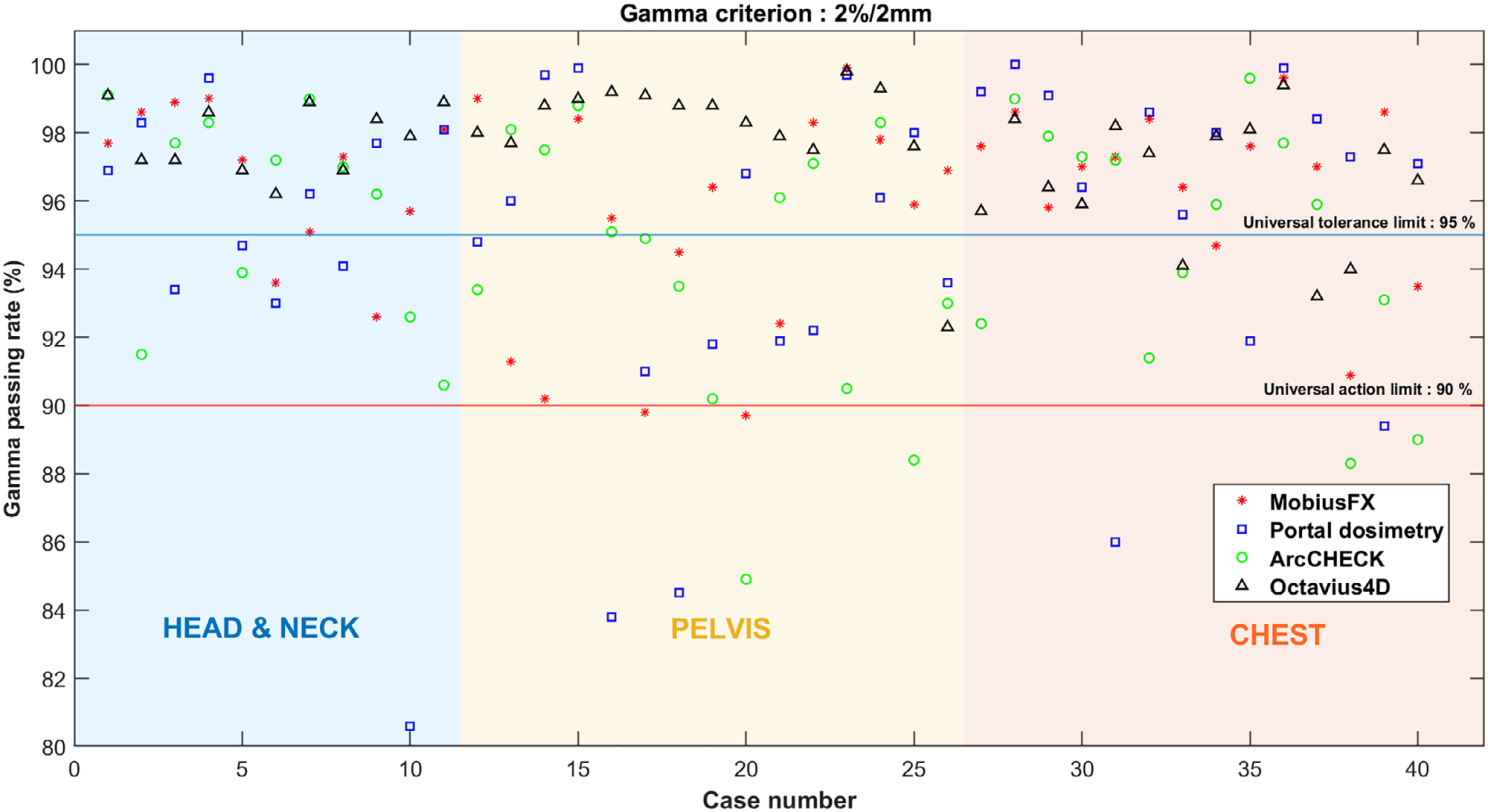
Comparison gamma passing rate across systems using the 2%/2 mm criterion. (The red line indicates the universal AL, and the blue line indicates the TL.) The gamma passing rate for each plan is represented using the following symbols: the asterisk (*) for MobiusFX, a square (□) for portal dosimetry, a circle (°) for ArcCHECK, and a triangle (△) for Octavius4D, respectively.

Figures 2, 3, 4 illustrate the plotted gamma passing rates against the universal TL (95%) and AL (90%) for the 3%/3 mm, 3%/2 mm, and 2%/2 mm criteria using both measurement‐based and log file‐based methods. When applying the 3%/3 mm criterion, all gamma passing rates obtained from both measurement‐based and log file‐based methods consistently exceeded 95% for all plans. Similarly, when the 3%/2 mm criterion was used, nearly all of the gamma passing rates obtained from both methods were higher than 95%, except for some of the gamma passing rates obtained by ArcCHECK, which were slightly below 95% but still above 90%. In contrast, when applying the strict 2%/2 mm criterion, the scatter plot illustrated a dispersion of passing and failing outcomes, indicating a lack of agreement between the systems. Therefore, the results indicated that the log file‐based method demonstrated strong concordance with the measurement‐based methods when applied to the 3%/3 mm and 3%/2 mm criteria. This concordance was evidenced by the consistent trend in which both methods produced passing rates exceeding the action and tolerance limits. Specifically, under the 3%/3 mm criterion, all plans achieved gamma pass rates exceeding the 95% threshold. Even under the more stringent 3%/2 mm criterion, both methods led to nearly the same clinical conclusion. A full dataset containing plan parameters and gamma passing rates for each system across all criteria is consolidated into a single comprehensive supplementary table (Table S1), which is available in the supplementary material.

In addition to presenting the graphical scatter plot, the percentage agreement scores were calculated across criteria, as demonstrated in Table 2. When employing either the 3%/3 mm or 3%/2 mm criteria, both systems achieved high percentage agreement scores, with the majority of the agreement scores reaching 100.00%. This was observed for both universal AL and TL. In contrast, when the 2%/2 mm criterion was utilized with either log file‐based or measurement‐based methods, it led to a decrease in the percentage agreement scores. Particularly, the percentage agreement scores sharply decreased when both systems were concurrently applied using the 2%/2 mm criterion. These findings also reflected that log file‐based method demonstrated good agreement with measurement‐based methods at the 3%/3 mm and 3%/2 mm criteria.

**TABLE 2:** The agreement scores between MobiusFX and measurement‐based QA (*n* = 40).

		Percentage agreement scores (%)		
		Log file ‐based method (MobiusFX)		
Gamma Criteria	Universal AL and TL for gamma passing rate	MobiusFX 3 % / 3 mm	MobiusFX 3 % / 2 mm	MobiusFX 2 % / 2 mm
Portal dosimetry 3 % / 3 mm	*Agreement cut‐off at 90 % (AL)*	*100.00*	*100.00*	*95.00*
	*Agreement cut‐off at 95 % (TL)*	*100.00*	*100.00*	*72.50*
Portal dosimetry 3 % / 2 mm	*Agreement cut‐off at 90 % (AL)*	*100.00*	*100.00*	*95.00*
	*Agreement cut‐off at 95 % (TL)*	*100.00*	*100.00*	*72.50*
Portal dosimetry 2 % / 2 mm	*Agreement cut‐off at 90 % (AL)*	*87.50*	*87.50*	*82.50*
	*Agreement cut‐off at 95 % (TL)*	*60.00*	*60.00*	*52.50*
ArcCHECK 3 % / 3 mm	*Agreement cut‐off at 90 % (AL)*	*100.00*	*100.00*	*95.00*
	*Agreement cut‐off at 95 % (TL)*	*100.00*	*100.00*	*72.50*
ArcCHECK 3 % / 2 mm	*Agreement cut‐off at 90 % (AL)*	*100.00*	*100.00*	*95.00*
	*Agreement cut‐off at 95 % (TL)*	*90.00*	*90.00*	*62.50*
ArcCHECK 2 % / 2 mm	*Agreement cut‐off at 90 % (AL)*	*90.00*	*90.00*	*90.00*
	*Agreement cut‐off at 95 % (TL)*	*55.00*	*55.00*	*52.50*
Octavius4D 3 % / 3 mm	*Agreement cut‐off at 90 % (AL)*	*100.00*	*100.00*	*95.00*
	*Agreement cut‐off at 95 % (TL)*	*100.00*	*100.00*	*72.50*
Octavius4D 3 % / 2 mm	*Agreement cut‐off at 90 % (AL)*	*100.00*	*100.00*	*95.00*
	*Agreement cut‐off at 95 % (TL)*	*100.00*	*100.00*	*72.50*
Octavius4D 2 % / 2 mm	*Agreement cut‐off at 90 % (AL)*	*100.00*	*100.00*	*95.00*
	*Agreement cut‐off at 95 % (TL)*	*87.50*	*87.50*	*70.00*

### 3.2 Investigation of RMS error and plan complexity

Table 3 presents the maximum RMS errors for each machine parameter across the 40 plans. There were no observed errors in the jaw, gantry angle, and collimator angle. However, subtle RMS errors were noticeable in the movement of the MLC for both banks A and B. The maximum RMS errors for banks A and B were 0.06 mm and 0.05 mm, respectively, across all individual leaves.

**TABLE 3:** The maximum RMS error in each machine parameter from 40 plans.

			Maximum RMS error of RMS error from all leaves (MLC)	
Maximum RMS error of Jaw (mm.)	Maximum RMS error of Collimator angle (°)	Maximum RMS error of Gantry angle (°)	Bank A (mm.)	Bank B (mm.)
Jaw X1 = 0 Jaw X2 = 0 Jaw Y1 = 0 Jaw Y2 = 0	0	0	0.06 ** minimum RMS error = 0.03	0.05 ** minimum RMS error = 0.03

**FIGURE 5:**
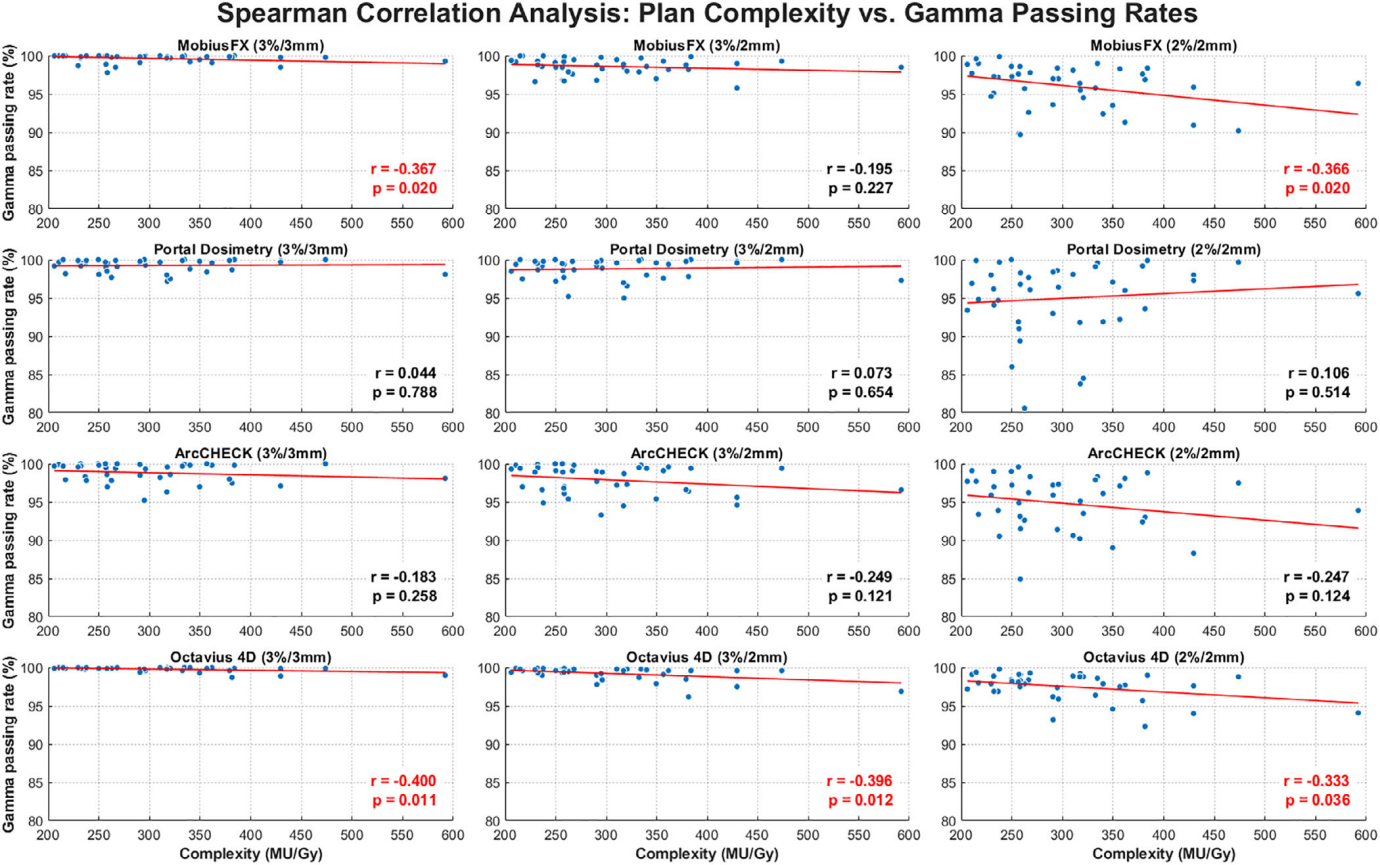
Spearman correlation analysis between MU/Gy and gamma passing rate for various measurement systems. The *r* values represent Spearman's rank correlation coefficients, and p denotes the statistical significance. Values highlighted in red indicate statistically significant correlations (*p* < 0.05).

To further investigate the relationship between the complexity metric and gamma passing rate in each system, additional analysis was carried out using Spearman's correlation test. The results are presented in Figure 5. Overall, a weak negative trend was observed between the complexity metric and gamma passing rate, although most correlations were not statistically significant. Only a few pairs reached statistical significance, with weak correlations in the log file‐based method (3%/3 mm and 2%/2 mm) and Octavius4D (3%/3 mm, 3%/2 mm, and 2%/2 mm), with correlation coefficients (*r*) of −0.367, −0.366, −0.400, −0.396, and −0.333, respectively. Consistent with these results, visual inspection of the scatter plots confirms the absence of a clear association, as the data points for gamma passing rate and MU/Gy are randomly scattered without an obvious trend or linear pattern.

### 3.3 Action and tolerance limits calculation

Table 4 demonstrates our local TLs and ALs for both log file‐based and measurement‐based methods. The local TLs for gamma passing rate with MobiusFX were 98.35%, 95.38%, and 88.38% for the 3%/3 mm, 3%/2 mm, and 2%/2 mm criteria, respectively. Additionally, for the same criteria, the local ALs were 98.10%, 94.75%, and 85.44%, respectively. The results illustrated that the TLs and ALs for the 3%/3 mm and 3%/2 mm criteria exceeded the universal criteria of 95% and 90%, respectively. On the other hand, the utilization of the 2%/2 mm criterion demonstrated that both the local TL and AL values fell below the universal recommendation (TG 218) for MobiusFX.

**TABLE 4:** Summarizing the local action and tolerance limits (AL and TL) for gamma passing rate in each system with 3%/3 mm, 3%/2 mm, and 2%/2 mm.

System	Gamma Criteria	Mean $\bar{X}\;$(%)	S.D. σ (%)	TL (%)	AL (%)
	3 % / 3 mm	99.65	0.53	98.35	98.10
MobiusFX	3 % / 2 mm	98.61	1.07	95.38	94.75
	2 % / 2 mm	96.07	2.85	88.38	85.44
	3 % / 3 mm	99.26	0.80	96.78	96.73
Portal dosimetry	3 % / 2 mm	98.82	1.31	94.82	94.71
	2 % / 2 mm	94.98	4.76	80.90	79.25
	3 % / 3 mm	98.82	1.24	95.74	94.87
ArcCHECK	3 % / 2 mm	97.89	1.87	92.95	91.54
	2 % / 2 mm	94.79	3.63	85.25	80.95
	3 % / 3 mm	99.80	0.32	99.12	98.87
Octavius4D	3 % / 2 mm	99.23	0.87	97.29	96.51
	2 % / 2 mm	97.53	1.80	93.34	90.82

Correspondingly, the gamma passing rates of the ALs and TLs for the measurement‐based methods were found to be higher than the universal recommendation for the 3%/3 mm and 3%/2 mm criteria. For applying the 3%/2 mm criterion, the TLs for portal dosimetry and ArcCHECK were found to be slightly lower than 95% by 0.18% and 2.05%, respectively. However, the ALs for portal dosimetry and ArcCHECK remained consistently higher than 90%. Similar to MobiusFX, when employing the 2%/2 mm criterion, the majority of local TLs and ALs in measurement‐based methods were found to be below the universal recommendation.

## 4 DISCUSSION

In this study, the log file‐based method (MobiusFX) was compared with measurement‐based methods for 40 VMAT patient plans. The results from both systems were compared in order to explore the possibility of implementing MobiusFX as an alternative approach for pretreatment verification QA. The study provides critical insights into the essential aspects of log file‐based PSQA, establishing a comprehensive framework to guide its clinical implementation and maximize its benefits for patient safety. The analyses specifically addressed the agreement between MobiusFX and measurement‐based methods, the relationships among RMS machine parameter errors, gamma passing rates, and plan complexity, as well as the establishment of action and tolerance limits for MobiusFX. Our findings have indicated that MobiusFX can produce interchangeable outcomes when compared to measurement‐based methods. Furthermore, we have also provided suggestions for the effective implementation in this study.

The growing interest in log file‐based PSQA is supported by multiple studies demonstrating its feasibility as an alternative pretreatment verification approach.^17^, ^18^, ^19^, ^20^, ^21^ In Basavatia et al.’s study,^17^ when applying the 3%/3 mm criterion, the gamma passing rate of MobiusFX demonstrated clinical comparability with various measurement‐based methods, including film dosimetry, EPID‐based dosimetry, and 2D ionization chamber arrays. Similarly, Vazquez‐Quino et al.’s study,^20^ also found that the MobiusFX system was comparable to ArcCHECK when using the 3%/3 mm criterion. Furthermore, Li and Price,^21^ have revealed that MobiusFX also matched well with measurement‐based PSQA at 3%/2 mm. Similar to our findings, it was revealed that using the 3%/3 mm and 3%/2 mm criteria with MobiusFX exhibited the high level of agreement with measurement‐based methods. The current study, along with the prior studies, demonstrated similar findings that strongly support the feasibility of utilizing log file‐based PSQA as the alternative method for pretreatment verification at the 3%/3 mm and 3%/2 mm criteria.

Beyond the conventional gamma analysis for PSQA analysis, the utilization of RMS error tolerance for machine parameters has been proposed in the log file‐based method. Chow et al.^12^ and Szeverinski et al.^13^ revealed that the use of these parameters effectively enhances the capability to detect and identify the source of errors. Therefore, it is noteworthy to consider the investigation of the RMS errors in this study. The maximum RMS errors for all machine parameters were zero, except for the subtle discrepancy observed in the MLC from both banks A and B, with 0.05 mm and 0.06 mm, respectively. In comparison to prior research, our study's maximum RMS errors for MLC were lower than the range of finding reported by Mubarok et al.^34^ In their study, the maximum RMS error for MLC was less than 0.8 mm for the Unique Varian linac machine. Additionally, McGarry et al. study,^35^ found maximum RMS errors for MLC to be 0.19, 0.46, and 0.45 for Varian TrueBeam and Clinacs (2300IX and 2100CD models), respectively. Furthermore, Agnew et al.^36^ found gantry position error in their TrueBeam linac, while we couldn't observe any gantry error movement in our machine. It has been discussed that the age of the machine and MLC performance might affect the machine's error.^35^, ^37^ The linac machine in this study has been used for only one year. Therefore, it might be the reason why machine errors were found to be negligible in this study. However, further investigation of the trend of RMS errors should be conducted to establish baselines for machine errors, which can contribute to determining tolerance levels in future log file‐based PSQA approaches.

It is crucial to recognize that machine errors are inherently unit‐specific and vary across institutions; these errors may be influenced by factors such as machine age and model, as discussed previously.^35^, ^36^, ^37^ Although no machine‐related errors were observed in the present study, log file‐based PSQA remains important for detecting deliverability errors arising from multiple sources, including plan transfer, machine performance, and human operation. Nevertheless, log file‐based PSQA (MobiusFX) has been shown to detect such discrepancies, consistent with previous research.^7^, ^11^, ^12^, ^13^ For instance, several studies that intentionally introduced machine errors, such as MLC and collimator positioning errors, demonstrated its high sensitivity capability for error detection.^7^, ^12^, ^13^ Furthermore, Rangaraj et al. performed log file analyses for 912 clinical plans, successfully identifying various issues, including data transfer errors and human‐related operational errors.^11^ These studies support the capability of log file‐based PSQA (MobiusFX) to reliably detect deliverability errors in clinical practice.

There has been a suggestion that the complexity of the plan might influence the gamma passing rate.^6^ Mohan et al.^38^ described that complex plans may include a large number of small apertures in MLC openings and MUs. These parameters could lead to a low gamma passing rate due to uncertainties in dose calculations associated with MLC modeling. Additionally, Kerns et al.^37^ explained that increasing these parameters could also be expected to increase leaf RMS errors. Our results revealed that there were weak or nonexistent correlations between complexity metric (MU/Gy) and gamma passing rate in each system (figure 5). The reasons might be that the treatment planning algorithm was already appropriately modeled. Moreover, the machine is currently performing well, and the MLC position errors were found to be negligible, as result from Table 3.

In this study, the local TL and AL of gamma passing rates were provided for both MobiusFX and measurement‐based PSQA in various gamma criteria. These limits were calculated by employing the statistical concept from TG 218.^6^ Our study showed that these local ALs and TLs might serve as helpful resources for establishing guidelines and comparing with other institutions that implement log file‐based PSQA. The findings indicated that, under the 3%/3 mm criterion, the TL and AL for MobiusFX were 98.35% and 98.10%, respectively. These values exceeded the standard limits (90% and 95%), suggesting the need to adopt more stringent criteria for MobiusFX. Furthermore, with the stricter 3%/2 mm criterion, the local TL and AL remained above the universal recommendations, with values of 95.38% and 94.75%, respectively. The TL and AL for the 3%/2 mm measurement‐based methods were also close to those of MobiusFX, indicating that both PSQA approaches follow a similar trend. Consequently, the same acceptance criteria could be applied to log file‐based PSQA in our institution. Compared to Li and Price study,^21^ which reported TL and AL ranges of 90–94% and 90–92% for MobiusFX using the 3%/2 mm criterion across four linear accelerators, the TL and AL values in the present study were notably higher. This likely reflects methodological differences: the previous study used patient CT images for calculating the dose distribution from log file, whereas the current study employed a homogeneous phantom (MVP) to eliminate inhomogeneity effects. The inclusion of inhomogeneity in the previous study may have affected dose differences between the TPS and Mobius3D calculations due to differences in the dose calculation algorithms, contributing to the lower gamma passing rates.

However, the implementation of MobiusFX as the alternative pretreatment verification method is not lacking in limitations or the need for caution. Agnew et al.^22^ found evident that the log file was unable to accurately record the position of the MLC due to the looseness of the T‐nut. Therefore, it is crucial to ensure the consistent maintenance of the MLC position and other machine parameters accurately, without any deviation from the baseline or tolerance. The test procedures and recommended tolerance limits can be adopted from TG 198.^39^ Furthermore, dosimetric parameters, such as beam output, are not included in the recorded data within the log file.^18^ Consequently, we highly recommend checking the beam output variation prior to employing the log file‐based PSQA. Additionally, the dose acquired through MobiusFX is calculated using its inherent algorithm and the vendor‐provided data of beam model. Hence, it is important to perform an appropriate commissioning process to investigate the accuracy of the vendor‐provided data. In particular, the accuracy of dose calculations in the Mobius3D can be greatly influenced by the configuration of the DLG correction factor, as illustrated by previous studies.^25^, ^40^ Therefore, adjustment of the DLG correction factor should be conducted prior to implementation. These elements are critical to ensuring the reliability and accuracy of log file‐based PSQA, as variations between measurement‐based systems may influence both the performance and clinical acceptance of MobiusFX.

## 5 CONCLUSION

Our study demonstrated that MobiusFX and measurement‐based methods yielded comparable and interchangeable gamma passing rates for both the 3%/3 mm and 3%/2 mm criteria, supporting the implementation of log file‐based PSQA as an alternative to measurement‐based pretreatment verification. Although RMS errors were not significant in this study, further investigation is warranted. The most appropriate TL and AL for MobiusFX were identified as 95.38% and 94.75%, respectively, at the 3%/2 mm criterion. In addition, successful clinical implementation requires careful consideration of machine‐ and system‐specific factors, including regular machine maintenance, beam output verification, and accurate beam model commissioning. These factors should be addressed prior to implementing MobiusFX in clinical practice to ensure reliability, accuracy, and clinical acceptance.

## AUTHOR CONTRIBUTIONS

**Thitipong Sawapabmongkon**: Writing—Original Draft; Visualization; Validation; Methodology; Investigation; Formal Analysis; Data Curation; Conceptualization. **Pimolpun Changkaew**: Methodology; Investigation; Validation; Formal Analysis; Data Curation; Writing—Review and Editing; Conceptualization. **Chanon Puttanawarut**: Validation; Investigation; Writing—Review and Editing. **Puangpen Tangboonduangjit**: Methodology; Investigation; Supervision; Writing—Review and Editing; Conceptualization. **Suphalak Khachonkham**: Writing—Original Draft; Visualization; Validation; Methodology; Investigation; Formal Analysis; Data Curation; Conceptualization; Writing—Review and Editing; Supervision; Project Administration.

## CONFLICT OF INTEREST STATEMENT

The authors have no relevant conflicts of interest to disclose.

## ETHICS STATEMENT

Ethical approval for this study was granted by the Institutional Review Board (IRB) of the Faculty of Medicine Ramathibodi Hospital, Mahidol University, Bangkok, Thailand (COA No. MURA2022/662).

## DECLARATIONS AND GENERATIVE AI Use

Artificial intelligence (AI) tools were used solely to assist with language editing, grammar correction, and the improvement of clarity. The scientific content, study design, data analysis, and interpretation of results were developed and carefully reviewed by the authors, who take full responsibility for the manuscript.

## Supporting information



Supporting Information
