# Supplementary material for: Implementing log file‐based patient‐specific QA for VMAT plans: A comparative study of MobiusFX and measurement‐based approaches
Source: J Appl Clin Med Phys. 2026 Feb 24;27(3):e70526. doi: 10.1002/acm2.70526 (PMC12931424; doi:10.1002/acm2.70526)
Supplement: Supplementary file 1 — Supporting Information [file ACM2-27-e70526-s001.docx]

| NO | **Energy** | **MU/Gy** | **No. Arc** | **Treatment size** | **log file-based PSQA (MobiusFX)** | | | **Portal dosimetry** | | | **ArcCHECK** | | | **Octavius4D** | | |
| --- | --- | --- | --- | --- | --- | --- | --- | --- | --- | --- | --- | --- | --- | --- | --- | --- |
|  |  |  |  |  | 3%/3 mm | 3%/2 mm | 2%/2 mm | 3%/3 mm | 3%/2 mm | 2%/2 mm | 3%/3 mm | 3%/2 mm | 2%/2 mm | 3%/3 mm | 3%/2 mm | 2%/2 mm |
| 1 | 6X | 210.5 | 3 | Head and neck | 100.00 | 99.20 | 97.70 | 99.70 | 99.40 | 96.90 | 99.90 | 99.80 | 99.10 | 100.00 | 99.90 | 99.10 |
| 2 | 6X | 258.5 | 3 | Head and neck | 100.00 | 99.90 | 98.60 | 99.70 | 99.50 | 98.30 | 98.50 | 97.00 | 91.50 | 100.00 | 99.90 | 99.10 |
| 3 | 6X | 206.0 | 3 | Head and neck | 100.00 | 99.40 | 98.90 | 99.20 | 98.50 | 93.40 | 99.70 | 99.30 | 97.70 | 99.90 | 99.40 | 97.20 |
| 4 | 6X | 334.5 | 3 | Head and neck | 100.00 | 99.70 | 99.00 | 100.00 | 100.00 | 99.60 | 99.90 | 99.80 | 98.30 | 100.00 | 99.80 | 98.60 |
| 5 | 6X | 236.5 | 2 | Head and neck | 100.00 | 98.60 | 97.20 | 99.50 | 99.20 | 94.70 | 98.30 | 96.60 | 93.90 | 99.80 | 99.00 | 96.90 |
| 6 | 6X | 290.5 | 3 | Head and neck | 99.10 | 96.80 | 93.60 | 99.50 | 99.20 | 93.00 | 99.90 | 99.00 | 97.20 | 99.80 | 99.00 | 96.20 |
| 7 | 6X | 232.0 | 3 | Head and neck | 99.70 | 99.30 | 95.10 | 99.60 | 99.50 | 96.20 | 100.00 | 99.90 | 99.00 | 100.00 | 99.90 | 98.90 |
| 8 | 6X | 232.2 | 3 | Head and neck | 99.90 | 98.80 | 97.30 | 99.20 | 98.70 | 94.10 | 99.70 | 99.50 | 97.00 | 99.90 | 99.30 | 96.90 |
| 9 | 6X | 266.5 | 3 | Head and neck | 98.50 | 97.60 | 92.60 | 99.90 | 99.80 | 97.70 | 99.40 | 99.10 | 96.20 | 100.00 | 99.80 | 98.40 |
| 10 | 6X | 262.5 | 3 | Head and neck | 99.80 | 97.90 | 95.70 | 97.70 | 95.20 | 80.60 | 97.80 | 95.40 | 92.60 | 99.90 | 99.50 | 97.90 |
| 11 | 6X | 310.4 | 3 | Head and neck | 100.00 | 99.50 | 98.10 | 99.70 | 99.60 | 98.10 | 98.20 | 97.20 | 90.60 | 100.00 | 99.80 | 98.90 |
| 12 | 6X | 217.0 | 3 | Pelvis region | 100.00 | 100.00 | 99.00 | 98.20 | 97.50 | 94.80 | 97.90 | 97.00 | 93.40 | 99.90 | 99.60 | 98.00 |
| 13 | 6X | 361.7 | 3 | Pelvis region | 99.10 | 98.20 | 91.30 | 99.60 | 99.40 | 96.00 | 99.80 | 99.60 | 98.10 | 100.00 | 99.60 | 97.70 |
| 14 | 6X FFF | 473.7 | 3 | Pelvis region | 99.80 | 99.30 | 90.20 | 100.00 | 100.00 | 99.70 | 100.00 | 99.40 | 97.50 | 99.90 | 99.60 | 98.80 |
| 15 | 6X FFF | 383.8 | 3 | Pelvis region | 100.00 | 99.90 | 98.40 | 100.00 | 100.00 | 99.90 | 99.80 | 99.40 | 98.80 | 99.90 | 99.60 | 99.00 |
| 16 | 10X | 317.6 | 3 | Pelvis region | 99.90 | 98.50 | 95.50 | 97.20 | 95.00 | 83.80 | 99.60 | 98.70 | 95.10 | 100.00 | 99.70 | 99.20 |
| 17 | 10X | 257.0 | 3 | Pelvis region | 98.90 | 96.80 | 89.80 | 99.00 | 98.60 | 91.00 | 99.50 | 98.90 | 94.90 | 100.00 | 99.90 | 99.10 |
| 18 | 10X | 320.6 | 3 | Pelvis region | 99.70 | 98.00 | 94.50 | 97.50 | 96.60 | 84.50 | 98.60 | 97.30 | 93.50 | 99.90 | 99.80 | 98.80 |
| 19 | 10X | 317.1 | 3 | Pelvis region | 99.70 | 98.90 | 96.40 | 98.00 | 97.00 | 91.80 | 96.30 | 94.50 | 90.20 | 99.80 | 99.60 | 98.80 |
| 20 | 6X | 258.3 | 3 | Pelvis region | 97.80 | 96.70 | 89.70 | 99.60 | 99.40 | 96.80 | 97.00 | 96.10 | 84.90 | 99.90 | 99.90 | 98.30 |
| 21 | 6X | 340.0 | 3 | Pelvis region | 99.20 | 98.60 | 92.40 | 98.80 | 98.00 | 91.90 | 99.80 | 99.40 | 96.10 | 100.00 | 99.70 | 97.90 |
| 22 | 6X | 356.6 | 3 | Pelvis region | 99.90 | 99.30 | 98.30 | 98.40 | 97.60 | 92.20 | 100.00 | 99.10 | 97.10 | 99.90 | 99.10 | 97.50 |
| 23 | 6X FFF | 237.7 | 2 | Pelvis region | 100.00 | 100.00 | 99.90 | 99.90 | 99.80 | 99.70 | 97.80 | 94.90 | 90.50 | 100.00 | 99.90 | 99.80 |
| 24 | 6X | 268.0 | 2 | Pelvis region | 99.90 | 99.50 | 97.80 | 99.10 | 98.70 | 96.10 | 100.00 | 99.80 | 98.30 | 100.00 | 99.80 | 99.30 |
| 25 | 6X | 429.4 | 3 | Pelvis region | 99.80 | 99.00 | 95.90 | 99.90 | 99.80 | 98.00 | 97.20 | 94.60 | 88.40 | 99.90 | 99.60 | 97.60 |
| 26 | 6X | 381.5 | 3 | Chest region (lung) | 99.80 | 98.20 | 96.90 | 98.70 | 97.80 | 93.60 | 97.50 | 96.40 | 93.00 | 98.70 | 96.20 | 92.30 |
| 27 | 6X | 379.0 | 4 | Chest region (lung) | 99.90 | 98.80 | 97.60 | 99.90 | 99.80 | 99.20 | 98.00 | 96.60 | 92.40 | 99.60 | 98.50 | 95.70 |
| 28 | 6X FFF | 249.5 | 3 | Chest region (lung) | 100.00 | 99.10 | 98.60 | 100.00 | 100.00 | 100.00 | 100.00 | 100.00 | 99.00 | 99.90 | 99.50 | 98.40 |
| 29 | 6X | 332.5 | 3 | Chest region (lung) | 99.90 | 97.90 | 95.80 | 99.90 | 99.90 | 99.10 | 99.70 | 99.50 | 97.90 | 99.60 | 98.70 | 96.40 |
| 30 | 6X | 296.0 | 3 | Chest region (lung) | 100.00 | 98.30 | 97.00 | 99.30 | 98.90 | 96.40 | 99.30 | 98.90 | 97.30 | 99.60 | 98.40 | 95.90 |
| 31 | 6X | 250.0 | 2 | Chest region (lung) | 100.00 | 98.50 | 97.30 | 98.10 | 97.20 | 86.00 | 99.80 | 99.10 | 97.20 | 99.90 | 99.60 | 98.20 |
| 32 | 6X FFF | 294.9 | 3 | Chest region (lung) | 99.90 | 99.80 | 98.40 | 100.00 | 99.90 | 98.60 | 95.20 | 93.30 | 91.40 | 99.80 | 99.20 | 97.40 |
| 33 | 6X | 592.0 | 4 | Chest region (lung) | 99.30 | 98.50 | 96.40 | 98.10 | 97.30 | 95.60 | 98.10 | 96.60 | 93.90 | 99.00 | 96.90 | 94.10 |
| 34 | 10X | 229.5 | 3 | Chest region (lung) | 98.70 | 96.60 | 94.70 | 99.90 | 99.80 | 98.00 | 99.60 | 98.90 | 95.90 | 100.00 | 99.70 | 97.90 |
| 35 | 6X | 256.5 | 3 | Chest region (Breast) | 100.00 | 98.50 | 97.60 | 99.70 | 99.50 | 91.90 | 100.00 | 100.00 | 99.60 | 99.90 | 99.30 | 98.10 |
| 36 | 6X FFF | 214.7 | 3 | Chest region (Breast) | 100.00 | 100.00 | 99.60 | 100.00 | 100.00 | 99.90 | 99.70 | 99.40 | 97.70 | 100.00 | 99.80 | 99.40 |
| 37 | 6X | 290.7 | 3 | Chest region (Breast) | 99.90 | 98.80 | 97.00 | 99.80 | 99.70 | 98.40 | 98.50 | 97.70 | 95.90 | 99.40 | 97.80 | 93.20 |
| 38 | 6X | 429.4 | 3 | Chest region (Breast) | 98.50 | 95.80 | 90.90 | 99.70 | 99.60 | 97.30 | 97.10 | 95.60 | 88.30 | 98.90 | 97.50 | 94.00 |
| 39 | 6X | 258.0 | 3 | Chest region (lung) | 100.00 | 99.20 | 98.60 | 98.50 | 97.70 | 89.40 | 98.60 | 96.80 | 93.10 | 99.90 | 99.40 | 97.50 |
| 40 | 6X | 349.5 | 2 | Chest region (Breast) | 99.50 | 97.00 | 93.50 | 99.80 | 99.60 | 97.10 | 97.00 | 95.40 | 89.00 | 99.30 | 97.90 | 94.60 |
| Mean | | | | | 99.65 | 98.61 | 96.07 | 99.26 | 98.82 | 94.98 | 98.82 | 97.89 | 94.79 | 99.80 | 99.23 | 97.53 |
| SD | | | | | 0.53 | 1.07 | 2.85 | 0.80 | 1.31 | 4.76 | 1.24 | 1.87 | 3.63 | 0.32 | 0.87 | 1.80 |
| Agreement scores percentage | | | | | MobiusFX  (3%/3mm) | *Agreement cut-off at 90 % (AL)* | | 100.00 | 100.00 | 87.50 | 100.00 | 100.00 | 90.00 | 100.00 | 100.00 | 100.00 |
|  |  |  |  |  |  | *Agreement cut-off at 95 % (TL)* | | 100.00 | 100.00 | 60.00 | 100.00 | 90.00 | 55.00 | 100.00 | 100.00 | 87.50 |
|  |  |  |  |  | MobiusFX  (3%/2mm) | *Agreement cut-off at 90 % (AL)* | | 100.00 | 100.00 | 87.50 | 100.00 | 100.00 | 90.00 | 100.00 | 100.00 | 100.00 |
|  |  |  |  |  |  | *Agreement cut-off at 95 % (TL)* | | 100.00 | 100.00 | 60.00 | 100.00 | 90.00 | 55.00 | 100.00 | 100.00 | 87.50 |
|  |  |  |  |  | MobiusFX  (2%/2mm) | *Agreement cut-off at 90 % (AL)* | | 95.00 | 95.00 | 82.50 | 95.00 | 95.00 | 90.00 | 95.00 | 95.00 | 95.00 |
|  |  |  |  |  |  | *Agreement cut-off at 95 % (TL)* | | 72.50 | 72.50 | 52.50 | 72.50 | 62.50 | 52.50 | 72.50 | 72.50 | 70.00 |

Table S1. Summary of patient plan parameters and gamma passing rates for 40 clinical VMAT cases, comparing MobiusFX with measurement-based systems (Portal Dosimetry, ArcCHECK, and Octavius4D) using 3%/3mm, 3%/2mm, and 2%/2mm criteria, including MobiusFX agreement scores percentage.
